# PADI3 plays an antitumor role via the Hsp90/CKS1 pathway in colon cancer

**DOI:** 10.1186/s12935-019-0999-3

**Published:** 2019-11-05

**Authors:** Zhengbin Chai, Li Wang, Yabing Zheng, Na Liang, Xiwei Wang, Yingying Zheng, Zhiwei Zhang, Chuanxi Zhao, Tingting Zhu, Chunyan Liu

**Affiliations:** 1 Medical Research Center, The First Affiliated Hospital of Shandong First Medical University, Jinan, 250014 China; 2 Shandong Provincial Key Laboratory for Rheumatic Disease and Translational Medicine, The First Affiliated Hospital of Shandong First Medical University, Jinan, 250014 China; 3 Department of Laboratory Medicine, Jinan Infectious Disease Hospital, Jingshi Road 22029, Jinan, 250021 Shandong People’s Republic of China; 4 Department of Obstetrics, The First Affiliated Hospital of Shandong First Medical University, Jinan, 250014 China; 5 Department of Traditional Chinese Medicine, The First Affiliated Hospital of Shandong First Medical University, Jinan, 250014 China; 6 Department of Anesthesiology, The First Affiliated Hospital of Shandong First Medical University, Jinan, 250014 China; 7 Shandong First Medical University & Shandong Academy of Medical Sciences, Jinan, 250014 China

**Keywords:** PADI3, Hsp90, CKS1, Colon cancer, Cell proliferation

## Abstract

**Background:**

CKS1 is highly expressed in colon cancer tissues, and is essential for cancer cell proliferation. The downstream molecular mechanism of CKS1 has been fully studied, but the upstream regulatory mechanism of it is still unclear. Earlier research found that PADI3 plays its anti-tumor roles via suppress cell proliferation, in this study, we found that the expression pattern of PADI3 and CKS1 are negatively correlated in colon cancer tissues, and overexpression of PADI3 can partly reverse CKS1 induced cancer cell proliferation. However, the regulatory mechanism of PADI3 and CKS1 in the tumorigenesis of colon cancer is still unclear and need to do further research.

**Methods:**

Western blot and real-time PCR were used to detect the expression levels of genes. CCK-8 and colony formation assays were used to examine cell proliferation and colony formation ability. Overexpression and rescue experiments were used to study the molecular mechanism of CKS1 in colon cancer cells, BALB/c nude mice were used to study the function of CKS1 in vivo.

**Results:**

CKS1 is highly expressed in colon cancer tissues, and the overexpression of CKS1 promotes cell proliferation and colony formation in both HCT116 (originating from primary colon cancer) and SW620 (originating from metastatic tumor nodules of colon cancer) cells. CKS1-expressing HCT116 cells produced larger tumors than the control cells. The expression pattern of PADI3 and CKS1 are negatively correlation in clinical samples of colon cancer, further study indicates that PADI3 can significantly decrease Hsp90 and CKS1 expression, and Hsp90 is essential for PADI3 to downregulate CKS1expression in colon cancer cells.

**Conclusions:**

PADI3 exerts its antitumor activity by inhibiting Hsp90 and CKS1 expression, and Hsp90 is essential for PADI3 to suppress CKS1 expression.

## Background

Colorectal cancer (CRC) is the third most common cancer and the fourth most common cause of cancer death. The incidence of CRC increases with age, and the risk is higher in men than in women [1]. Genetic and environmental factors are the major contributors to CRC development [2]. The loss of cell cycle control is one of the major causes of tumorigenesis, further studies on the molecular mechanisms of cell cycle control are essential for the prevention and therapy of colon cancer.

Cyclin-dependent kinase regulatory subunits (CKSs) can interact with Cyclin-dependent kinases (CDKs) to regulate the cell cycle [3]. There are two CKSs exist in mammalian cells, named CKS1 and CKS2 [4]. CKS1 and CKS2 are essential for cell proliferation, for CKS1 and CKS2 double-mutated mice can be lethal [5]. However, increasing evidences have shown that CKS1 has high expression level in various cancers, such as colorectal carcinoma, lung cancer, prostate carcinoma, breast cancer, lymphomas and take part in tumorigenesis as an oncogene [6–10]. The inhibition of CKS1 expression is important for cell cycle control in tumor cells, and it is considered to be a very promising therapy for cancer. Further study the molecular mechanism of CKS1 in tumorigenesis and find out the suppressor of it is meaningful for anti-cancer drug design and cancer therapy.

Heat shock protein 90 (Hsp90) is a well-known molecular chaperone, and hundreds of proteins have been identified to interact with it [11]. Hsp90 can participate in various important physiological processes by regulating the stability, function and activity of numerous proteins [12]. Earlier reports showed that inhibiting Hsp90 can relieve CKS1-induced drug resistance and progression in cancer therapy [13, 14]. Increasing evidences have shown that inhibitors of Hsp90, such as AUY922, 17-AAG, TAS-116, and NCT-50, have significant antitumor activity [15–19]. According to the previously study, we speculate that Hsp90 and CKS1 may crosstalk with each other to take part in tumorigenesis, which still need further study.

Although the downstream signal pathway of both CKS1 and Hsp90 were well studied, the upstream regulating mechanism and relationship of CKS1 and Hsp90 in the tumorigenesis of colon cancer was little known. Further study the function of CKS1 and Hsp90 in the tumorigenesis of colon cancer and search for the inhibitors of them sounds to be useful for colon cancer therapy.

Protein arginine deiminases (PADs) can catalyze the conversion of arginine residues to citrulline residues in the presence of excess calcium, which plays an important role in tumorigenesis [20–22]. In our previous study, we found that PADI3, which belongs to the PADs family, can inhibit cell proliferation via inducing G1-phase arrest to play its antitumor role in colon cancer [23]. In the present study, we found that the expression pattern of PADI3, CKS1 and Hsp90 are negative correlation in colon cancer tissues, and overexpression of PADI3 can partly reverse CKS1 induced cell proliferation and colony formation. We speculated that PADI3 may plays its anti-tumor activity via suppress CKS1 induced cell proliferation. However, the molecular mechanism of it still needs further explore.

In this study, we found that CKS1 has a higher expression level in colon cancer tissues, overexpression of CKS1 can promote colon cancer cell proliferation and elevate the colony formation ratio in both HCT116 and SW620 cells. CKS1-expressing HCT116 cells can promote tumor growth in vivo. A molecular mechanism study showed that the Hsp90 inhibitor 17-AAG can suppress CKS1 and CDK1 expression and promote p27^kip1^ expression, while PADI3 can suppress Hsp90 expression. Further research showed that PADI3 can suppress CKS1 and CDK1 expression and promote p27^kip1^ expression, which is consistent with the effect of Hsp90 inhibitor 17-AAG. Rescue experiments indicated that both PADI3 and 17-AAG can partly reverse the CKS1 overexpression-induced cell proliferation and colony formation. The overexpression of PADI3 can induce the inhibition of cell proliferation and colony formation in colon cancer cells. Moreover, PADI3 suppressed CKS1 and CDK1 expression and promoted p27^kip1^ expression can be partly reversed by Hsp90 overexpression.

These findings suggest that the CKS1 expression-induced cell proliferation and colony formation can be blocked by PADI3 via downregulating Hsp90 expression in colon cancer cells and that PADI3 is a promising inhibitor of Hsp90 as a tumor suppressor gene.

## Methods

### Ethics

BALB/c nude mice were housed in an Association for Assessment and Accreditation of Laboratory Animal Care (AAALAC)-accredited facility. The study was approved by the Ethics Committee of Shandong Province Qianfoshan Hospital, Jinan, China (Approval number: S0087).

### Inclusion criteria of clinical samples and tissue collection

The patients information including gender, age (20 < adults < 60 years old), pathological stage are clear and available, primary colon cancer tissues diagnosed by pathology, and there is available corresponding adjacent tissue as control.

All solid colon cancer tissues and adjacent tissues used in this study were obtained from patient surgeries at the Shandong Provincial Qianfoshan Hospital in Jinan, Shandong, P. R. China. Tumor diagnosis was performed via histological methods, and pathological categorization was performed according to the World Health Organization (WHO) classification system.

### Antibodies information

The following primary antibodies used in this study were commercially obtained: PADI3 (Abcam, ab172959), GAPDH (Abcam, ab181603), His-tag (CST, 12698S), Flag (CST, 14793S), red fluorescent protein (RFP) (Abcam, ab28664), CKS1 (ThermoFisher, 36-6800), CDK1 (ThermoFisher, 33-1800), Hsp90 (CST, 4874S), and p27^Kip1^ (CST, 3686S). The following secondary antibodies were used in this study: goat anti-rabbit (Affinity, s0001), goat anti-mouse (Affinity, s0002) and donkey anti-goat (Abcam, ab6881).

### Quantitative real-time-PCR analysis

Total RNA of tissues or cells was extracted using Trizol reagent (Life Technologies, USA). The extracted RNA was reverse-transcribed into first-strand cDNA in a final volume of 10 µL using an RNA PCR Kit (Toyobo, Japan). The reverse-transcribed first-strand cDNA was used as the template for real-time PCR with the forward primer and the reverse primer. The conditions of real-time PCR were as follows: 10 s at 95 °C; 45 cycles of 5 s at 60 °C and 10 s at 72 °C; and 30 s at 65 °C. This experiment was performed in triplicate. Real-time PCR was performed using a 10 µL total volume that contained 1 µL of cDNA, 2 µL of ddH_2_O, 5 µL of SYBR Green Real-time PCR Master Mix (Toyobo, Japan), 1 µL of forward primer and 1 µL of reverse primer. GAPDH was used for quantity and quality control using the forward primer of human GAPDH-QF and the reverse primer of human GAPDH-QR. The primer sequences in detail used in this study are shown in Additional file 1: Table S1. Data were analyzed using the formula R = 2^−[ΔCt sample − ΔCt control]^, where R is the relative expression level, ΔCt sample is the difference between the Ct of the gene and the average GAPDH in the experiment sample, and ΔCt control is the difference between the Ct of the gene and the average GAPDH in the control sample.

### Western blot analysis

One hundred micrograms of tissue were homogenized in 500 μL of cell lysis buffer (KeyGEN BioTECH, China) and centrifuged at 12,000 rpm for 15 min at 4 °C. The supernatant was collected as the total protein, and the protein concentrations were determined using the Bradford Protein Assay Kit (Beyotime, China). About 30 μg total proteins for each sample was loaded and separated by 12.5% or 10% sodium dodecyl sulfate polyacrylamide gel electrophoresis (SDS-PAGE) and transferred onto a 0.22 μm aperture polyvinylidene fluoride (PVDF) membrane (Millipore, USA). The PVDF membrane was then incubated with blocking buffer (5% skim milk powder dissolved in TBS) for about 1 h at room temperature. After washed thrice with TBST (10 mM Tris–HCl, pH of 7.5, 150 mM NaCl, and 0.05% Tween-20), the PVDF membrane was then incubated with primary antibody (1:1000 in Primary Antibody Dilution Buffer (Beyotime, China)) for 16 h or overnight at 4 °C. After washed thrice with TBST, the PVDF membrane was then incubated with HRP-conjugated secondary antibody (1:5000 in blocking buffer) for about 1 h at room temperature. The PVDF membrane was then washed thrice with TBST, and the acquisition of enhanced chemiluminescence (ECL) images was carried out with the Typhoon Trio System (GE Healthcare, USA).

### Construction of plasmids

#### Construction of CKS1 recombinant plasmid

The full open reading frame (ORF) of CKS1 is 240 bp and encodes 79 amino acid residues. The ORF of CKS1 was amplified by PCR using the CKS1(h)-OE-EcoRI-Fex and CKS1(h)-OE-AscI-Rex primers (Primer sequences are shown in Additional file 1: Table S1). The PCR products were inserted into pCDNA3.1-RFP expression vectors using the restriction enzymes *EcoR*I and *Asc*I, which contains a His-tag and are resistant to ampicillin. RFP was selected as the reporter gene and was inserted into multiple cloning sites (MCSs) using the restriction enzymes *Kpn*I and *Not*I. Recombinant CKS1-expressing plasmids were sequenced by BGI (Beijing, China), purified using the GeneJET Plasmid Miniprep Kit (ThermoFisher, USA) and stored at − 80 °C.

#### Construction of PADI3 recombinant plasmid

The full ORF of PADI3 is 1995 bp and encodes 664 amino acid residues. PADI3 cDNA was synthesized by YouBio (Changsha, China). PADI3 was amplified by PCR using the PADI3-EcoRI-Fex and PADI3-AscI-Rex primers. The PCR products were inserted into pCDNA3.1-RFP expression vectors using the restriction enzymes *EcoR*I and *Asc*I, which contain a His-tag and are resistant to ampicillin. RFP was selected as the reporter gene and was inserted into MCSs using the restriction enzymes *Kpn*I and *Not*I. Recombinant PADI3-expressing plasmids were sequenced by BGI (Beijing, China), purified using the GeneJET Plasmid Miniprep Kit (ThermoFisher, USA) and stored at − 80 °C.

#### Construction of Hsp90 recombinant plasmid

The full ORF of Hsp90 is 2199 bp and encodes 732 amino acid residues. Hsp90 cDNA was obtained from Genechem (Genechem, Shanghai, China). Hsp90 was amplified by PCR using the Hsp90-*Not*I-Fex and Hsp90-*Kpn*I-Rex primers. The PCR products were inserted into p3 **× **FLAG-CMV-7.1 expression vectors using the restriction enzymes *Not*I and *Kpn*I, which contains a FLAG-tag and are resistant to ampicillin. Green fluorescent protein (GFP) was amplified by PCR using the GFP-*Kpn*I-Fex and GFP-*Sma*I-Rex primers and inserted into p3 **× **FLAG-CMV-7.1-Hsp90 using the restriction enzymes *Kpn*I and *Sma*I as the reporter gene. The GFP-expressing recombinant plasmids p3 × FLAG-CMV-7.1-GFP was selected as the negative control. The recombinant plasmids were purified using the GeneJET Plasmid Miniprep Kit (ThermoFisher, USA) and stored at − 80 °C.

### PADI3 gene structural prediction analysis

The PADI3 sequence was obtained from GenBank (https://www.ncbi.nlm.nih.gov/genbank/). Sequence analysis was performed using BLASTX software (http://www.ncbi.nlm.nih.gov/). Gene translation and protein prediction were performed using ExPASy (http://www.au.expasy.org/).

### Lentivirus-coated expression plasmids

#### Lentivirus-coated CKS1-expressing plasmid

The full ORF of CKS1 was inserted into Ubi-MCS-3FLAG-CBh-gcGFP-IRES-Puro expression vectors and coated with lentivirus by Genechem (Genechem, Shanghai, China). The lentivirus-coated Ubi-CKS1-3FLAG-CBh-gcGFP-IRES-Puro recombinant plasmids were transfected into HCT116 cells and screened with 2 μg/mL puromycin. Ubi-MCS-3FLAG-CBh-gcGFP-IRES-Puro was transfected into HCT116 cells as the control group.

#### Lentivirus-coated PADI3-expressing plasmid

The full coding sequence of PADI3 was inserted into pHBLV-CMVIE-T2A-Puro lentiviral vectors containing the RFP reporter gene (Hanbio, China). Recombinant pHBLV-CMVIE-PADI3-T2A-Puro viruses were transfected into HCT116 cells and screened with 2 μg/mL puromycin. pHBLV-CMVIE-RFP-T2A-Puro was transfected into HCT116 cells as the control group.

### Cell proliferation assay

Cell proliferation assays were performed using a Cell Counting Kit-8 (Dojindo, Japan). The recombinant plasmids were transfected into SW620 or HCT116 cells using PolyJet™ DNA In Vitro Transfection Reagent (SignaGen, USA) according to the manufacturer’s instructions. After culturing for 12 h, the cell culture medium was discarded, and 100 μL of fresh complete culture medium (containing 10% FBS and a 1% penicillin/streptomycin solution) was added and separately cultured for another 12 h, 24 h or 48 h at 37 °C with 5% CO_2_. Then, the CCK-8 solution (Dojindo, Japan) at a concentration of 10 μL/well was added into the cell culture medium, and culturing was continued for another 2 h. The absorbance was measured with a spectrophotometer at 450 nm (SpectraMax 190, Molecular Device, USA). Graphs showing cell growth were generated from the average values of five wells in each group, and data were obtained from 3 independent experiments.

### Cell colony formation assay

#### For HCT116 cells

HCT116 cells were transfected with recombinant plasmids as described above. After culturing for 48 h, the cells were harvested, and 1000 cells in 6 mL of complete culture medium were seeded in a 6-cm-diameter petri dish. After 10 days of culturing, colonies were fixed with methanol and stained with 0.25% crystal violet. The number and size of the colonies were analyzed from three independent experiments.

#### For SW620 cells

SW620 cells were transfected with recombinant plasmids as described above. After culturing for 48 h, the cells were harvested, and 3000 cells in 6 mL of complete DMEM were seeded in a 6-cm-diameter petri dish. After 14 days of culturing, colonies were fixed with methanol and stained with 0.25% crystal violet. The number and size of the colonies were analyzed from three independent experiments.

### Establishment of tumor-bearing mice with HCT116 cells

The full coding sequence of CKS1 was inserted into Ubi-CKS1-3FLAG-CBh-gcGFP-IRES-Puro lentiviral vectors containing the GFP reporter gene (Genechem, Shanghai, China). Recombinant Ubi-CKS1-3FLAG-CBh-gcGFP-IRES-Puro viruses were transfected into HCT116 cells and screened with 2 μg/mL puromycin. Ubi-MCS-3FLAG-CBh-gcGFP-IRES-Puro was transfected into HCT116 cells as the control group. Twelve BALB/c nude mice at 6 weeks old (Vital River, China) were randomly divided into two groups to establish the tumor-bearing mouse model. Two hundred microliters of CKS1-expressing HCT116 cells (10^7^/mL in phosphate-buffered saline (PBS)) were injected into the dorsal flank of each mouse in the experimental group, and 200 µL of GFP-expressing HCT116 cells (10^7^/mL in phosphate-buffered saline (PBS)) were injected into the dorsal flank of each mouse in the control group. Tumors were dissected at 42 days after cell injection; the size and weight of the tumors were measured by routine methods. This experiment was repeated three times independently.

### Statistics

Data were analyzed by a two-tailed Student’s *t* test. Differences were considered to be statistically significant at p < 0.05. To verify the results, each experiment was performed with three samples in triplicate.

## Results

### CKS1 is highly expressed in colon cancer tissues

**Fig. 1:**
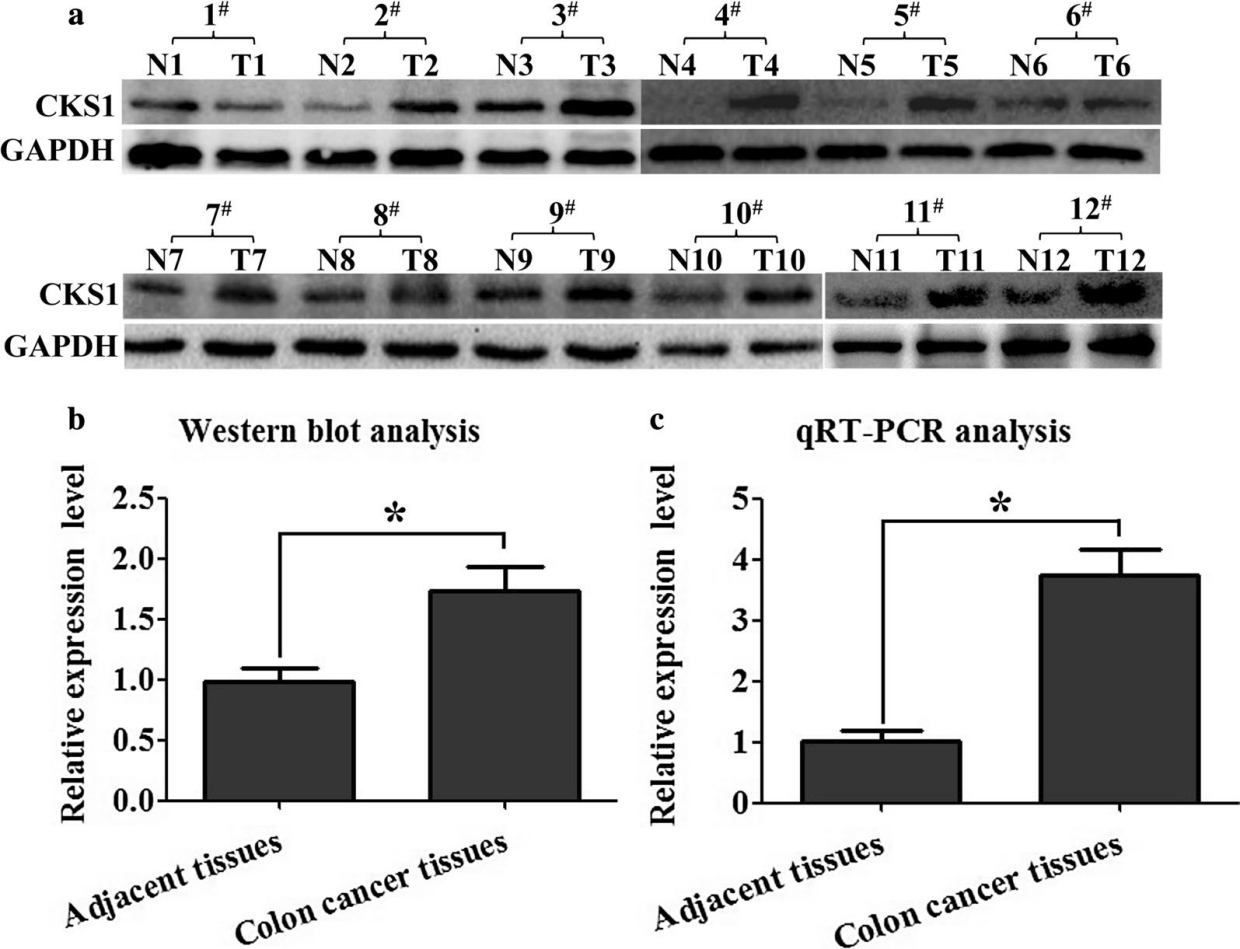
Expression profile of CKS1 in colon cancer and their corresponding adjacent tissues determined using qRT-PCR and Western blot analysis. **a** Western blot analysis was used to measure the expression level of CKS1 in colon cancer tissues and their corresponding adjacent tissues at the translational level. These paired tissue samples were obtained from 12 different patients; GAPDH was used to normalize the relative expression level of CKS1; **b** statistical analysis of Western blot; **c** qRT-PCR was used to measure the expression level of CKS1 in the colon cancer tissues and corresponding adjacent tissues at the transcriptional level. N: corresponding adjacent tissues, T: tumor tissues. *Indicates p < 0.05 for three independent experiments analyzed by Student’s t test

To fully study the function of CKS1 in colon cancer, the expression profile of it was examined using western blot and qRT-PCR in colon cancer tissues and their corresponding adjacent tissues which were obtained from 12 different patients. Results showed that there was only a little expression of CKS1 in the adjacent tissues. However, a high expression level of CKS1 was detected in the corresponding colon cancer tissues both in translational level (Fig. 1a, b) and in transcriptional level (Fig. 1c). This finding suggests that CKS1 mainly expressed in colon cancer tissues and may play an important role in the tumorigenesis of colon cancer.

### CKS1 promotes colon cancer cell proliferation and colony formation

**Fig. 2:**
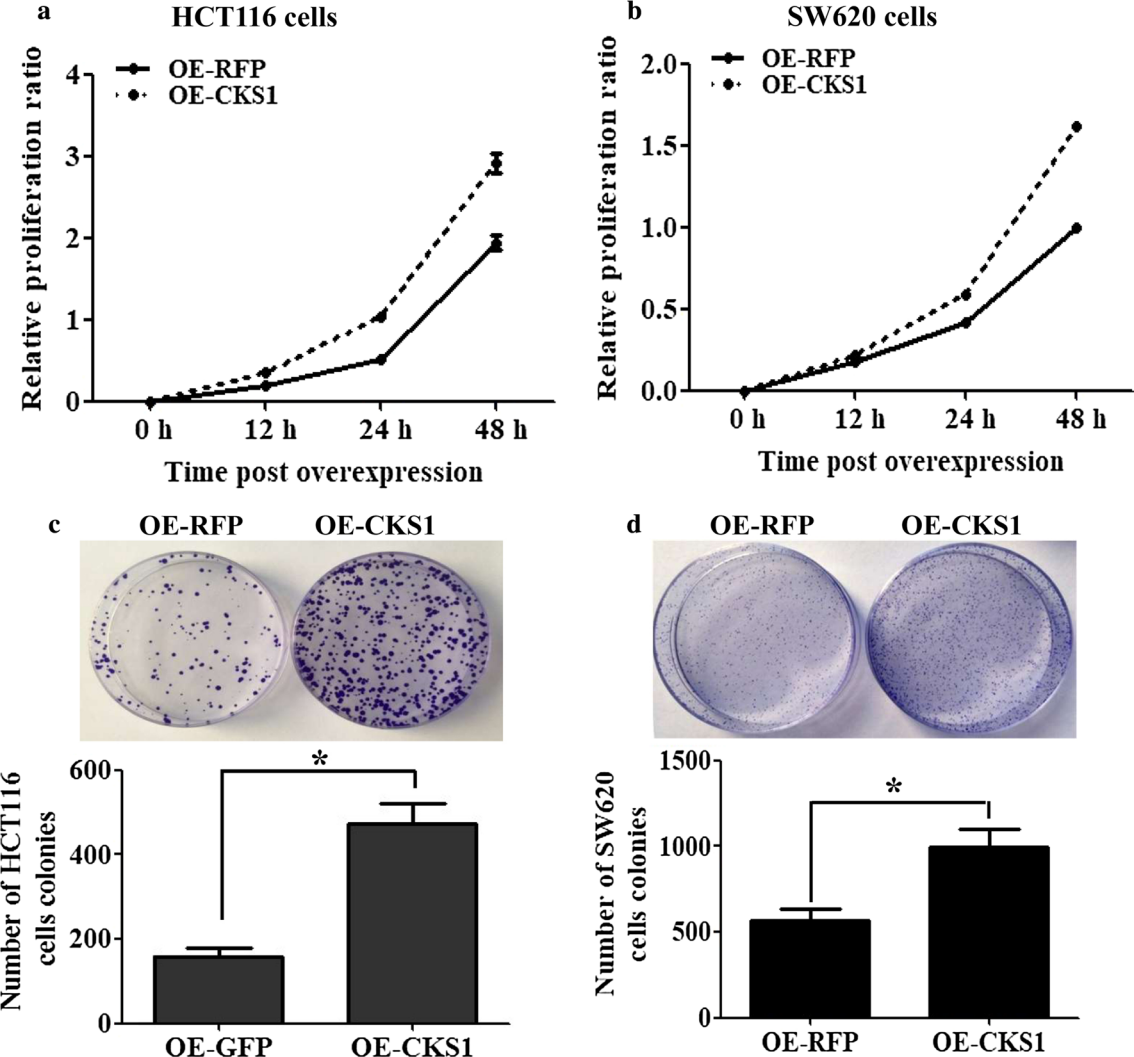
Function of CKS1 in HCT116 and SW620 cells. pCDNA3.1-CKS1-RFP plasmids were transfected to HCT116 and SW620 cells to study the function of CKS1 in colon cancer cells, pCDNA3.1-RFP plasmids transfected cells were used as controls; **a** CCK-8 assay was used to measure the proliferation ratio of HCT116 cells post plasmid was transfected for 12 h, 24 h and 48 h, respectively; **b** CCK-8 assay was used to measure the proliferation ratio of SW620 cells post plasmid was transfected for 12 h, 24 h and 48 h, respectively; **c** the colony formation ability of HCT116 cells was measured and statistically analyzed using a colony formation assay following 10 days of culture; **d** the colony formation ability of SW620 cells was measured and statistically analyzed using a colony formation assay following 14 days of culture. *Indicates p < 0.05 for three independent experiments analyzed by Student’s t test

To investigate the role of CKS1 in colon cancer, CKS1 was transfected into HCT116 cells and SW620 cells to study the proliferation ratio and colony formation activity. RFP was transfected into HCT116 cells and SW620 cells, separately, as the controls. The results showed that in both the HCT116 cells and SW620 cells, CKS1-overexpressing cells had a higher cell proliferation activity (Fig. 2a, b) and colony formation ability (Fig. 2c, d) than the control groups. These results indicate that CKS1 may take part in tumorigenesis of colon cancer via promoting cell proliferation and colony formation in vitro.

### CKS1-overexpressing HCT116 cells promote tumor growth in vivo

**Fig. 3:**
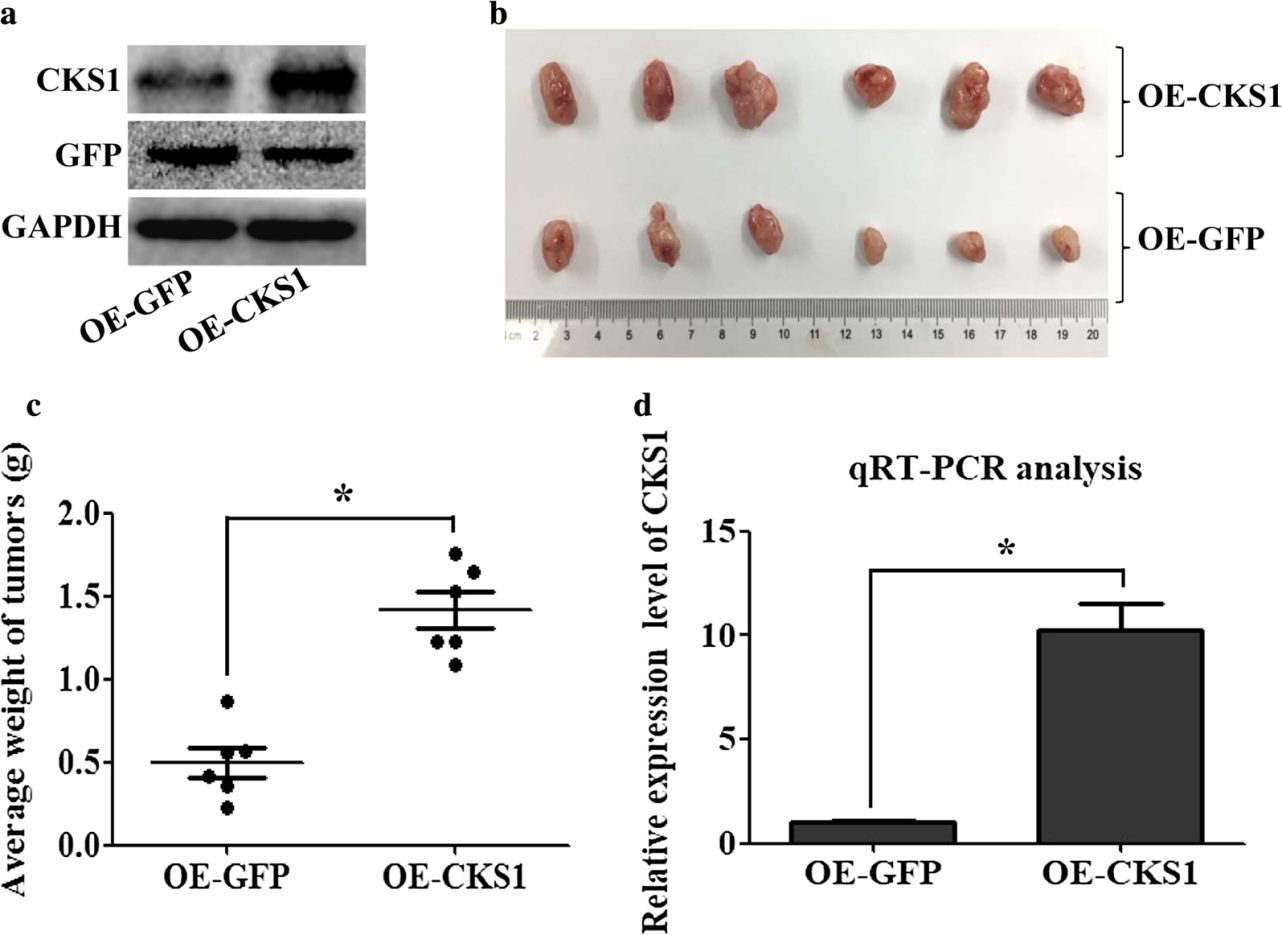
Function study of CKS1 in vivo. CKS1-expressing HCT116 cells were injected into the BALB/c nude mice to study the function of CKS1 in tumorigenesis, GFP-expressing HCT116 cells were injected into the BALB/c nude mice as the control group; **a** western blot analysis was used to confirm CKS1 expression level both in CKS1-expressing HCT116 cells and in GFP-expressing HCT116 cells; **b** tumors were dissected 42 days after cell injection, the upper group of tumors was developed from CKS1-expressing HCT116 cells, and the lower group of tumors was developed from GFP-expressing HCT116 cells; **c** tumors weight were measured and statistically analyzed; **d** qRT-PCR was used to measure the expression level of CKS1 in the tumor tissues dissected after 42 days injection. *Indicates p < 0.05 for three independent experiments analyzed by Student’s t test

To further study the function of CKS1 in colon cancer, lentiviral-coated CKS1 was transfected into HCT116 cells, and this CKS1-overexpressing HCT116 cells were injected into 6-week-old BALB/c nude mice, whereas GFP-overexpressing HCT116 cells was used to inject 6-week-old BALB/c nude mice as the control group. Results showed that lentiviral-coated CKS1 was transfected into HCT116 cells successfully (Fig. 3a), and this CKS1-expressing HCT116 cells can significantly promote tumor growth in vivo (Fig. 3b, c), these larger tumor tissues obtained from experiment group has a high expression level of CKS1 than tissues obtained from control group (Fig. 3d). This finding indicates that high level of CKS1 can promote colon cancer tumor growth in vivo.

### Both 17-AAG and PADI3 can suppress CKS1 expression

Earlier reports have shown that the Hsp90 inhibitor 17-AAG can suppress CKS1, CDK1 expression, promote p27^kip1^ expression and lead to G1-phase arrest in colon cancer cells. Our previous study found that PADI3 can inhibit cell proliferation via inducing G1-phase arrest. According to that, we speculate that PADI3 may play its anti-tumor role via affect Hsp90, CKS1, CDK1 and p27^kip1^ expression in colon cancer.

**Fig. 4:**
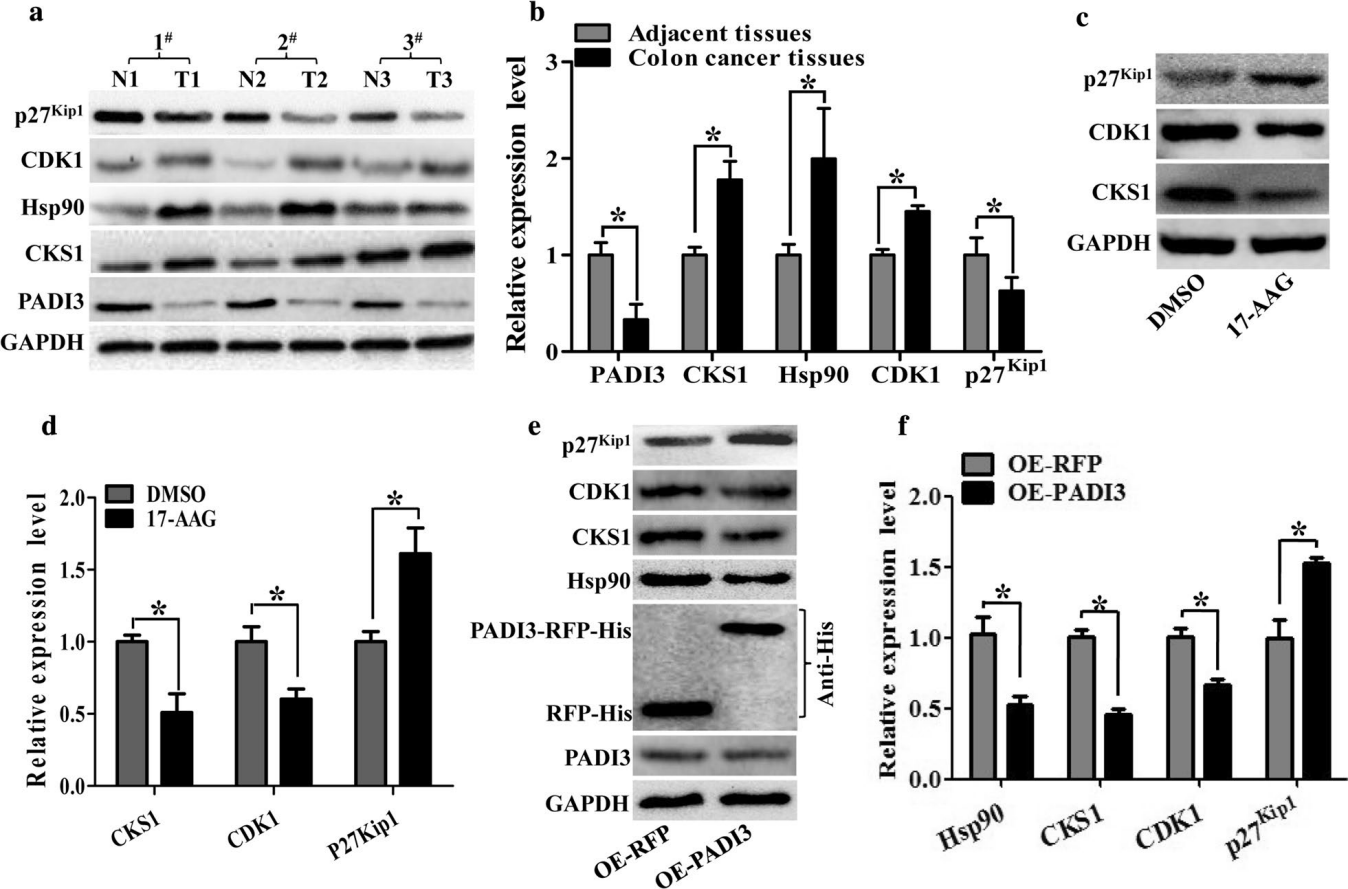
Both 17-AAG and PADI3 can suppress CKS1 expression in HCT116 cells. **a** Expression profile of PADI3, CKS1, Hsp90, CDK1 and p27^kip1^ were detected using western blot in colon cancer tissues and their corresponding adjacent tissues at the translational level, GAPDH was used to normalize the relative expression of them; **b** statistical analysis of western blot results in A; **c** 5 μM 17-AAG was used to treat HCT116 cells for 24 h, and western blot was used to measure the expression level of CKS1, CDK1 and p27^kip1^; **d** Statistic analysis of **c**; **e** Overexpression of PADI3 was performed in HCT116 cells, and western blot was used to detect the expression level of Hsp90, CKS1, CDK1 and p27^kip1^, overexpression of RFP as the control group; **f** statistical analysis of western blot results in **e**. GAPDH was selected as the internal control, *indicates p < 0.05 for three independent experiments analyzed by Student’s t test

To verify these results, we detected the expression pattern of PADI3, Hsp90, CKS1, CDK1 and p27^kip1^ in colon cancer tissues and their corresponding adjacent tissues, results showed that both PADI3 and p27^kip1^ had lower expression level in colon cancer tissues than in the corresponding adjacent tissues. In contrast, Hsp90, CKS1 and CDK1 had higher expression level in colon cancer tissues than in the corresponding adjacent tissues (Fig. 4a, b). Further study found that 17-AAG can suppress CKS1 and CDK1 expression and promote p27kip1 expression in HCT116 cells, which agree with the earlier study (Fig. 4c, d).

To determine whether PADI3 has a similar function as 17-AAG, we transfected HCT116 cells with PADI3-overexpressing plasmids and measured the expression levels of Hsp90, CDK1, CKS1 and p27^kip1^. Results showed that PADI3 functions similar to 17-AAG, which can inhibit Hsp90, CDK1, CKS1 expression and promote p27^kip1^ expression (Fig. 4e, f) These results indicate that PADI3 may be a promising inhibitor of both Hsp90 and CKS1.

### PADI3 blocks CKS1 expression-induced cell proliferation and colony formation

**Fig. 5:**
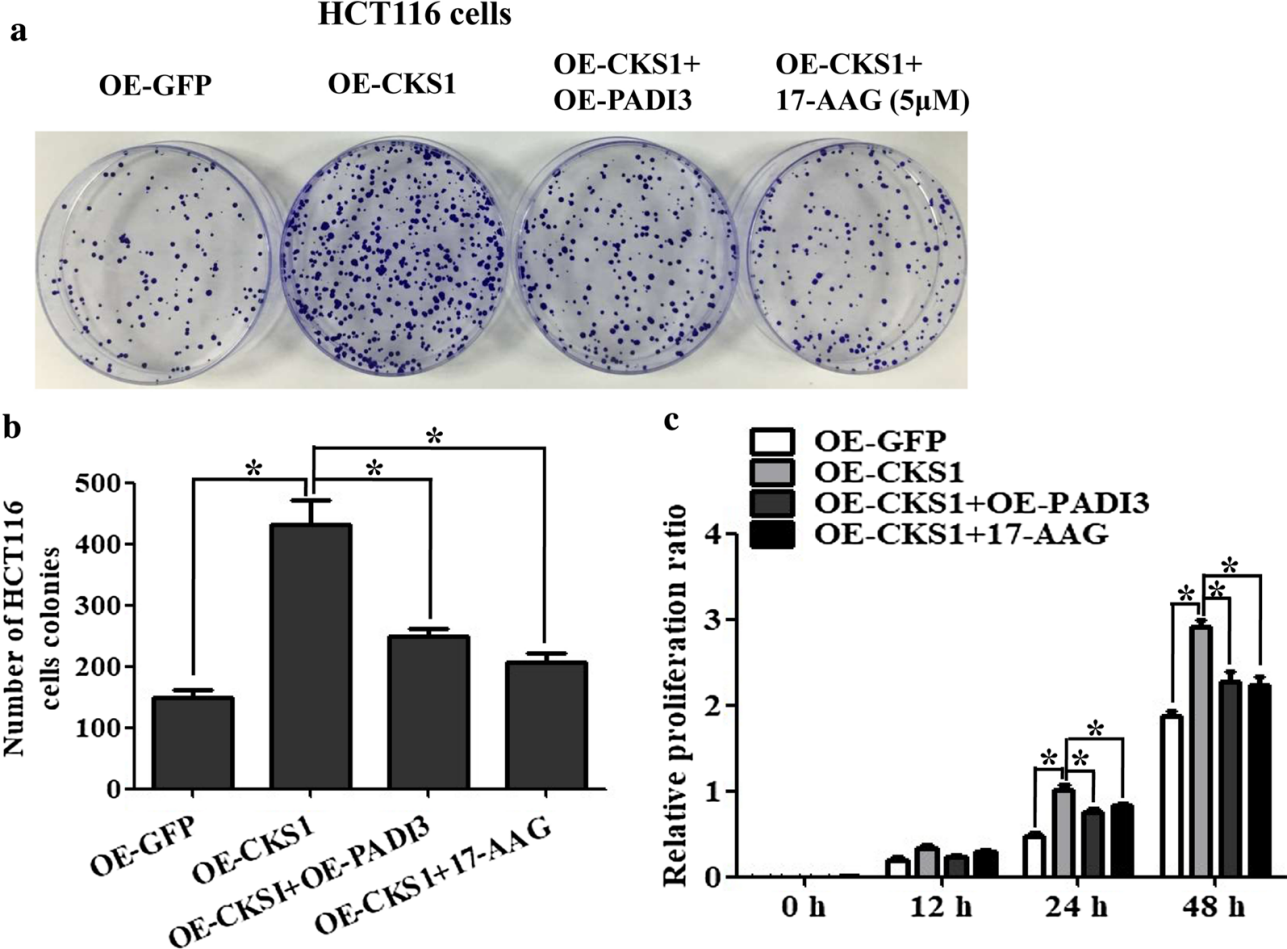
Function of PADI3 on CKS1 overexpression induced cell colony formation and proliferation in HCT116 cells. **a** GFP stable expressing HCT116 cells as the negative control, CKS1stable expressing HCT116 cells as the positive control, PADI3 overexpression plasmid was transfected into the CKS1 stable expressing HCT116 cells to study the function of PADI3 on CKS1 induced cell colony formation activity, 5 μM 17-AAG was used to treat CKS1stable expressing HCT116 cells to study the function of 17-AAG on CKS1 induced cell colony formation activity; **b** statistic analysis of colony formation results in **a**; **c** CCK8 was used to study the function of PADI3 and 17-AAG on CKS1 induced cell proliferation at 0 h, 12 h, 24 h and 48 h. *Indicates p < 0.05 for three independent experiments analyzed by Student’s t test

To verify the function of PADI3 in CKS1-induced tumorigenesis in vitro, cell proliferation and colony formation activity were measured. The results showed that the transfection of the PADI3 overexpression plasmid into lentivirus-transfected CKS1-stably expressed HCT116 cells blocked CKS1 expression-induced colony formation (Fig. 5a, b)and cell proliferation (Fig. 5c), which is similar to the function of 17-AAG. These results indicate that PADI3 can block CKS1 overexpression induced colon cancer cell colony formation and proliferation ability in vitro.

### PADI3 inhibits CKS1 expression via downregulating Hsp90

**Fig. 6:**
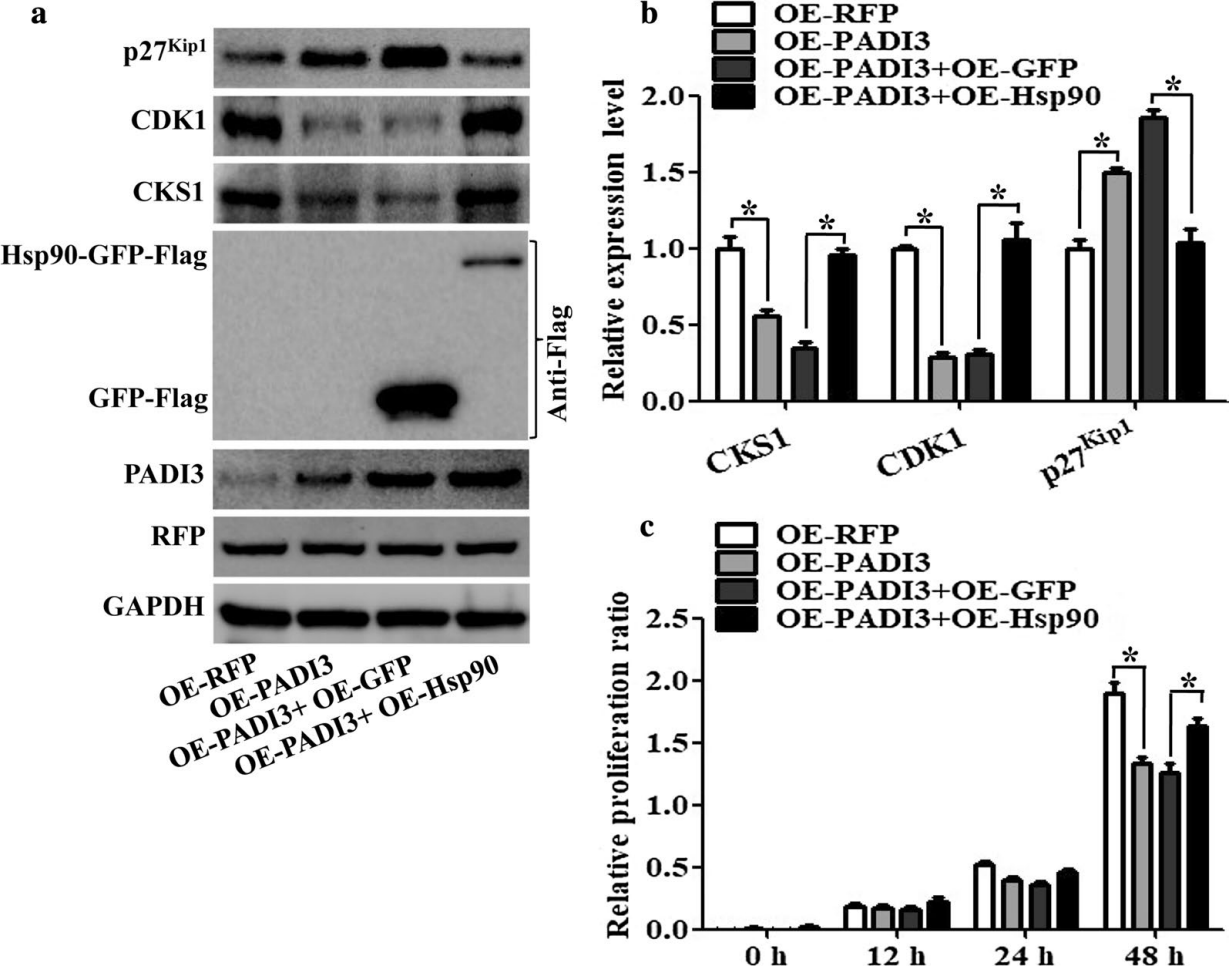
Function of Hsp90 on PADI3 regulated CKS1 expression. RFP stable expressing HCT116 cells as the negative control, PADI3 stable expressing HCT116 cells as the positive control, Hsp90 overexpression plasmid was transfected into the PADI3 stable expressing HCT116 cells to study the function of Hsp90 on PADI3 regulated CKS1 expression, GFP overexpression plasmid was transfected into the PADI3 stable expressing HCT116 cells as the negative control group. **a** Western blot was used to measure the expression level of CKS1, CDK1 and p27kip1 after Hsp90 transfected into PADI3 stable expressing HCT116 cells; **b** qRT-PCR was used to verify the results of **a**. **c** CCK8 analysis was used to study the function of Hsp90 in the regulating of cell proliferation in PADI3 stable expressing HCT116 cells. GAPDH was selected as the internal control, *indicates p < 0.05 for three independent experiments analyzed by Student’s t test

To further study the molecular mechanism of PADI3 in regulating CKS1, we transfected HCT116 cells with a lentivirus-coated PADI3-expressing plasmid and screened using puromycin (2 μg/mL) to establish a PADI3-stably expressing HCT116 cell line. The Hsp90-expressing plasmid was then transfected into the PADI3-stably expressing HCT116 cells. Results showed that overexpression of Hsp90 can practically recover the PADI3-affected CKS1, CDK1 and p27^kip1^ expression both in translational level (Fig. 6a) and in transcriptional level (Fig. 6b). CCK-8 analysis was also performed to verify the results, which showed that the overexpression of Hsp90 in PADI3-stably expressing HCT116 cells can overcome PADI3-suppressed cell proliferation (Fig. 6c).

## Discussion

### CKS1 promotes cell proliferation, colony formation and tumor growth in colon cancer

CKS1 is essential for cell proliferation via regulating the cell cycle transition from the G1 to the S phase [24], plays an important role in tumorigenesis as an oncogene, and previous research suggests that inhibiting CKS1 expression may be an excellent strategy for cancer therapy [7, 25]. An increasing number of studies have found that CKS1 is highly expressed in various malignant tumors. However, the function and molecular mechanism of CKS1 in colon cancer is still unclear. In this study, we found that CKS1 has a higher expression level in colon cancer tissues than in their corresponding adjacent tissues. In vitro, the overexpression of CKS1 can promote cell proliferation and colony formation in both SW620 cells and HCT116 cells. In vivo, CKS1-overexpressing HCT116 cells injected into BALB/c nude mice led to larger tumors than those in the controls, and these results agree with the results of previous studies which showed that CKS1 contributes to tumorigenesis in colon cancer [26, 27]. The suppression of CKS1 expression may be a beneficial strategy for colon cancer therapy.

### Inhibiting Hsp90 expression can decrease CKS1 expression

Hsp90 is well known as an oncogene, and a high expression level of Hsp90 is associated with decreased survival and poor prognosis in various cancers [28–30]. Inhibiting Hsp90 expression has been considered to be a promising antitumor treatment [31–33]. Previous studies found that inhibitors of Hsp90 can block cell proliferation, but the molecular mechanism is still unclear. In this study, we found that 17-AAG, an Hsp90 inhibitor, can suppress CKS1 and CDK1 expression and promote p27^kip1^ expression. CKS1, CDK1, and p27^kip1^ are essential for cell cycle control and cell proliferation [34, 35]. Thus, inhibit Hsp90 expression can downregulate CKS1 expression and lead to the suppression of cell proliferation in colon cancer cells.

### PADI3 plays an antitumor role by regulating Hsp90/CKS1 expression

The discovery of Hsp90 and CKS1 inhibitors with low toxicity and high efficiency is essential for colon cancer therapy. Our previous study showed that PADI3 plays an antitumor role in colon cancer by suppressing cell proliferation and cell colony formation, but the molecular mechanism is still unclear. In this study, we found that PADI3 exerts its antitumor activity by suppressing Hsp90 and CKS1 expression, and Hsp90 is essential for PADI3 to suppress CKS1 expression. Although the key functional domain and sites of PADI3 in this process require further exploration in future studies.

## Conclusion

We found that PADI3 is an effective inhibitor of Hsp90 and CKS1 for regulating the cell cycle in colon cancer cells and that Hsp90 is essential for PADI3 to downregulate the expression of CKS1.

## Supplementary information

**Additional file 1: Table S1.** PCR primer sequences.

## Data Availability

All data generated or analyzed during this study are included in this published article and its additonal files.
