# Supplementary material for: PADI3 plays an antitumor role via the Hsp90/CKS1 pathway in colon cancer
Source: Cancer Cell Int. 2019 Nov 5;19:277. doi: 10.1186/s12935-019-0999-3 (PMC6833139; doi:10.1186/s12935-019-0999-3)
Supplement: Supplementary file 1 — Additional file 1: Table S1. PCR primer sequences. [file 12935_2019_999_MOESM1_ESM.docx]

| Primer | Sequence |
| --- | --- |
| CKS1(h)-OE-EcoRI-Fex: | TACTCAGAATTCATGTCGCACAAACAAATT |
| CKS1(h)-OE-AscI-Rex: | TACTCAGGCGCGCCGATTTCTTTGGTTTCTTGGG |
| PADI3-EcoRI-Fex： | TACTCAGAATTCATGTCGCTGCAGAGAATC |
| PADI3-AscI-Rex： | TACTCAGGCGCGCCGAGGGCACCATGTTCCACCA |
| Hsp90-NotI-Fex： | TACTCAGCGGCCGCGATGCCTGAGGAAACCCAG |
| Hsp90-KpnI-Rex： | TACTCAGGTACCGAGTCTACTTCTTCCATGCG |
| GFP-KpnI-Fex： | TACTCAGGTACCAATGAGCAAGGGCGAGGAA |
| GFP-SmaI-Rex： | TACTCACCCGGGATCACTTGTACAGCTCGTC |
| CKS1(h)-QF1： | TATTCGGACAAATACGACGACG |
| CKS1(h)-QR1： | CGCCAAGATTCCTCCATTCAGA |
| Hsp90(h)-QF1: | GCTTGACCAATGACTGGGAAG |
| Hsp90(h)-QR1: | AGCTCCTCACAGTTATCCATGA |
| PADI3-QF: | CCCTCGTGGACATTTATGGGT |
| PADI3-QR: | ATCTCCAAAGTCGCGTCAAAG |
| GAPDH-QF: | GCACCGTCAAGGCTGAGAAC |
| GAPDH-QR: | TGGTGAAGACGCCAGTGGA |

**Table S1. PCR primer sequences**
